# Supplementary material for: Content-rich biological network constructed by mining PubMed abstracts
Source: BMC Bioinformatics. 2004 Oct 8;5:147. doi: 10.1186/1471-2105-5-147 (PMC528731; doi:10.1186/1471-2105-5-147)
Supplement: Additional File 2 — The original results of the above study (non-essential files are deleted to keep the file size under the limit set by BMC bioinformatics). [file 1471-2105-5-147-S2.bz2 › chilibotAdditionalFile2/dip40/426ID11470772E10356/html/CCR1_CCL1.html]

 


 **CCR1** and **CCL1** 
  
Found 34 abstracts in PubMed, retrieved 34.  
 

 What does Google say? 
 PDF only 
| .edu only 

---

**Interactive relationship** (e.g. stimulation, inhibition, etc)

**Neutral relationship**- All of these data support the conclusion that I 309  [ **CCL1** ]  is a functional ligand for CKR  [ **CCR1** ]  L1.  Ref: 9469461 J Immunol, 1998
- Natural proteolytic processing of hemofiltrate CC chemokine 1  [ **CCL1** ]  generates a potent CC chemokine receptor CCR 1  [ **CCR1** ]  and CCR5 agonist with anti HIV properties.  Ref: 11085751 J Exp Med, 2000
- These data demonstrate that TER1 ChemR1 or CKR  [ **CCR1** ]  L1 is the receptor for I 309  [ **CCL1** ] , and we propose to call this receptor CCR8 in agreement with the current nomenclature for chemokine receptors.  Ref: 9211859 J Biol Chem, 1997
- Proteolytic processing of the abundant plasmatic human CC chemokine 1  [ **CCL1** ]  HCC 1 generates a truncated form, HCC 1 9 74, which is a potent agonist of  **CCR1** , CCR3, and CCR5.  Ref: 11897579 Antimicrob Agents Chemother, 2002
- The CC chemokine receptor CCR  [ **CCR1** ]  8 belongs to the seven transmembrane spanning receptor family and functionally responds to the eukaryotic CC chemokines I 309  [ **CCL1** ] , thymus and activation regulated chemokine, macrophage inflammatory protein 1 beta MIP 1b and to the products viral MIP I and viral MIP II of the Kaposi associated herpesvirus HHV 8.  Ref: 10611408 Forum Genova < MedlineTA>Forum Genova , 2002

**Non-interactive relationship** (e.g. studied together, co-existance, homology, etc.)

- Second, renal clearance of para aminohippurate CPAH, inulin CIn, creatinine CCr  [ **CCR1** ] , free water CH2O and chloride CCl  [ **CCL1** ]  was evaluated in 7 untreated patients aged 17 44 yrs, 4 males and 3 females and in 7 controls aged 24 39 yrs, 5 males and 2 females.  Ref: 1868921 Nippon Naibunpi Gakkai Zasshi, 1991
- To test the in vivo relevance of this, we used a murine melanoma system and knockout mice to investigate the function of the chemokine receptor CCR5 and its ligands, CCR  [ **CCR1** ]  ligand CCL  [ **CCL1** ]  3 and CCL5.  Ref: 12682253 J Immunol, 2003
- The most active chemokines for resting Th IL 10, in terms of calcium mobilization and in vitro migration, were in order of potency CCL2 monocyte chemoattractant protein 1, CCR2 ligand, CCL4 macrophage inflammatory protein 1beta, CCR5 ligand, CCL3 macrophage inflammatory protein 1alpha,  **CCR1**  5 ligand, CCL17 thymus and activation regulated chemokine, CCR4 ligand,  **CCL1**  I 309  [ **CCL1** ] , CCR8 ligand, CXCL12 stromal derived factor 1, CXCR4, and CCL11 eotaxin, CCR3 ligand.  Ref: 11145678 J Immunol, 2001
- ACZ but not FRU decreased CCr  [ **CCR1** ] , despite lesser increases of V, CLi and CH2O CCl  [ **CCL1** ]  rough indexes of distal delivery.  Ref: 7712144 Exp NephrolExp Nephrol, 2001
- Renal function urine volume UV, 24 h excretion of Na, K and Cl UNA V, UK V, UCl V, clearance of Na CNa, K CK and Cl CCl  [ **CCL1** ] , creatinine CCr  [ **CCR1** ] , osmotic substances Cosm, and solute free water FWC, and the fractional excretion of Na, K and Cl and ALDO were measured for an additional 3 consecutive days during the training period.  Ref: 12405745 Equine Vet J Suppl, 2002
- There were no significant changes in heart rate, daily urinary volume, sodium balance, and renal clearances of sodium CNa, potassium CK, chloride CCl  [ **CCL1** ]  and creatinine CCr  [ **CCR1** ] .  Ref: 7047789 Jpn Circ J, 1982
- Nevertheless, intermediate maturity DCs expressed substantial levels of Flt 3L, IGF 1, IL 1alpha and 1beta, IL 6, CCL  [ **CCL1** ] , 1 7, and 22, MIP 2, osteopontin, CCR  [ **CCR1** ] , 5, and 7, and CXCR 4.  Ref: 11902830 Cell Immunol, 2001
- The detection of the CC chemokine receptor CCR  [ **CCR1** ]  8 message in human umbilical vein endothelial cells HUVECs by reverse transcription polymerase chain reaction RT PCR and RNase protection assay RPA, prompted us to investigate the potential role exerted by the CC chemokine I 309  [ **CCL1** ] , a known ligand of such receptor, in both in vitro and in vivo angiogenesis assays.  Ref: 11110671 Blood, 2000
- As expected for chemokine receptor mediated effects, pertussis toxin, but not cholera toxin, inhibited both the calcium flux and migration of the CKR  [ **CCR1** ]  L1 transfectants in response to I 309  [ **CCL1** ] .  Ref: 9469461 J Immunol, 1998
- Furthermore, TCA3  [ **CCL1** ]  does not induce a Ca2 lux in cells transfected with cDNA for the C C CKR 1  [ **CCR1** ]  chemokine receptor, supporting the conclusion that there are distinct receptors for TCA3  [ **CCL1** ] .  Ref: 7963534 J Immunol, 1994
- CCL  [ **CCL1** ]  receptors, CCR2,  **CCR1** , and CCR5, also showed increased expression in mdx muscle.  Ref: 12609504 Neuromuscul Disord, 2003
- We have previously isolated from human hemofiltrate an N terminally truncated form of the hemofiltrate CC chemokine 1  [ **CCL1** ]  HCC 1, and characterized HCC 1 9 74 as a strong agonist of  **CCR1** , CCR5, and to a lower extent CCR3.  Ref: 11544332 J Immunol, 2001
- CCr  [ **CCR1** ]  and CCl  [ **CCL1** ]  P less than 0.05.  Ref: 6524740 Am J Vet Res, 1984
- Changes in CCr  [ **CCR1** ] , V, CNa, CCl  [ **CCL1** ] , CH2O, CLi were studied before and after acute administration of acetazolamide ACZ to 11 normal subjects or FRU to 9 normal subjects.  Ref: 7712144 Exp NephrolExp Nephrol, 1984
